# Are cultured mesenchymal stromal cells an option for immunomodulation in transplantation?

**DOI:** 10.3389/fimmu.2013.00041

**Published:** 2013-02-26

**Authors:** Jan A. Plock, Jonas T. Schnider, Riccardo Schweizer, Vijay S. Gorantla

**Affiliations:** ^1^Division of Plastic and Hand Surgery, University Hospital ZurichZurich, Switzerland; ^2^Department of Plastic Surgery, University of Pittsburgh Medical Center, University of PittsburghPittsburgh, PA, USA

Mesenchymal stromal cells (MSCs) are under investigation for clinical application. Despite approval by the United States Food and Drug Administration for MSC use in pediatric steroid-refractory acute GvHD after allogeneic hematopoietic stem cell transplantation (Parekkadan and Milwid, 2010) uncertainty about the fate of MSCs after infusion remains thus far. Clinical trials have provided evidence for high response rates, efficacy, and safety leading to mortality reduction after MSC treatment of GvHD (Bernardo et al., 2010). Recently de Girolamo et al. (2012) have reviewed clinical observations and critical aspects extensively. Systemic immunomodulation following MSC treatment has been demonstrated (Dander et al., 2012; Zanotti et al., 2013), regardless of the many unresolved questions including possible entrapment in the lungs and liver, homing to sites of inflammation or trauma, and the relevance of chimerism.

Recently, Eggenhofer et al. (2012) presented evidence in a mouse model that cultured bone-marrow derived MSCs are entrapped in the lungs after intravenous infusion. These results confirm that, in addition to a need for greater understanding of their functional and immunologic characteristics, there is also a need to investigate the migratory properties of cultured MSCs in circulation prior to clinical implementation. We congratulate Eggenhofer et al. for their experimental insights. Herein, we propose to summarize some salient aspects of existing literature evidence and our own experience in response to some comments and conclusions made by the authors.

The present study supports the findings from Fischer et al. (2009), who have described a first-pass effect in the lung capillaries for MSCs. These authors used MSCs up to passage 4 and could demonstrate that cells from a second bolus injection passed the lungs more efficiently. This study elegantly could show a dependency of MSCs and their ability to pass the lung filter on size and surface antigens.

In rodent and swine transplant models, intravenous delivery of MSCs has been shown to achieve long-term peripheral blood chimerism. Some of these studies (Kuo et al., 2009, 2011; Pan et al., 2010) prove that MSCs survived for months or long-term in the periphery without complete entrapment in the pulmonary capillary bed. However, they also confirmed on histopathology that homing of MSCs to lungs does occur (Kuo et al., 2009).

**Figure 1:**
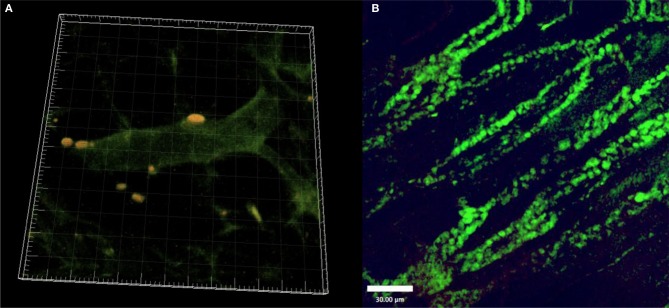
**(A)** Fluorescent MSC (yellow) after perivascular homing to critically ischemic skin. 3D reconstruction from Laser-Scanning Confocal Microscopy. **(B)** Fluorescent MSCs (green) 14 days after tailvein injection and homing to critically ischemic skin. (Freshly isolated BM-MSC; tail vein injection).

In our own experiments, we investigated sites of vascular regeneration in a critically perfused skin-flap model in immunocompetent mice (C57BL/6) after transplantation of fluorescent allogeneic MSCs. Freshly isolated Lin^−^CD105^+^ bone-marrow derived MSCs (2 × 10^5^/animal in 100 μm 0.9% NaCl via tail vein injection), were infused via tail vein injection. MSCs exhibited perivascular homing *remote* to the lungs and liver as well as paracrine expression of growth factors mediating vascular regeneration in specific sites. We were able to visualize MSCs *in vivo* by intravital fluorescence microscopy and laser scanning confocal microscopy and *post mortem* histologically in the peripheral tissue (Schlosser et al., 2012). Over time, cell numbers increased but they did not change their morphology (Figure 1). Yet, we could not differentiate whether this was due to local proliferation or further recruitment of MSCs in these experiments.

Cultured MSCs may not be phenotypically distinguishable from fibroblasts and may even share similar surface antigens or differentiation potential (Hematti, 2012). With regards to cultured fibroblasts, we found that these cells induced lethal pulmonary embolism if infused too quickly (<1 min; own unpublished data) but slow IV injection was consistent with survival. The findings by Eggenhofer et al. (2012) of predominant lung entrapment explain our observational findings of mortality. However, in other studies, Schlosser et al. (2012) reported that entrapped fibroblasts exhibit regenerative effects over critically perfused skin. These findings may indirectly support conclusions of Eggenhofer et al. that MSCs could mediate distant effects via endocrine mechanisms.

In the past, it has been shown that the duration and degree of cell expansion and culture has a clear impact on MSC morphology, differentiation, viability, and migratory properties (Wagner et al., 2010). Freshly isolated MSCs show superior homing ability compared to expanded cells (Rombouts and Ploemacher, 2003), which might be due to their size (own unpublished data; Fischer et al., 2009) as well as unique homing factors. Importantly, MSCs not only undergo phenotypic changes in culture and during passage (size, morphology, and cell surface marker expression) (Wagner et al., 2010; Hematti, 2012), but also lose capacity for functional proliferation and differentiation potential (Vacanti et al., 2005; Wagner et al., 2010). In addition, their ability for cytokine production is altered (Banfi et al., 2002; Vacanti et al., 2005).

To avoid the first pass effect and consequent pulmonary capillary entrapment following MSC transplantation, Zonta et al. (2010) suggest an arterial route of access. They delivered MSCs to the renal artery during kidney transplantation in rodents and reported favorable recovery of kidney function as opposed to the intravenous route. Arterial application might thus enable direct delivery to the capillary bed of the graft with reduced cell loss through entrapment and consequent unwarranted systemic effects.

Pulmonary and hepatic entrapment of MSCs has been intensely debated and studied for years. The study by Eggenhofer et al. is the first to lucidly demonstrate that cultured MSCs undergo significant entrapment in the lung after intravenous application. It still remains speculative: (1) If the degree of this phenomenon varies with the size of MSCs infused (based on passage cycle or culture denominators); (2) if there are long-term effects on lung function due to the entrapped cells and; (3) if the immunological efficacy of MSCs could be improved through direct arterial delivery to the graft or specific end organs. There is some evidence that the loss of cells through a first pass effect is indeed lower with freshly isolated MSCs indicating a link to smaller cell size or possibly related to enhanced viability and homing capacity.

Taken together, studies comparing effects of fresh isolated MSCs delivered intra-arterially to the graft or in proximity to the end organ to those secondary to passaged MSCs delivered via a peripheral intravenous route may be important to define if indeed this is a technical or procedural consideration essential for incorporation into pre-clinical protocols to optimize overall outcomes.
